# Research progress on the compositional characteristics of the tumor immune microenvironment and immunopredictive models in esophageal squamous cell carcinoma

**DOI:** 10.1080/15384047.2026.2641263

**Published:** 2026-03-11

**Authors:** Gongcheng Wang, Zhenzhen Li, Siyu Tan, Jing Ma, Zhiyong Yin, Juan Du, Linni Fan

**Affiliations:** a Basic Medical Research and Experiment Center, School of Medicine, Yan'an University, Yan'an, China; b Department of Pathology, State Key Laboratory of Cancer Biology, Xijing Hospital, Air Force Military Medical University, Xi'an, China; c Department of Cardiology, Xijing Hospital, Air Force Military Medical University, Xi'an, China

**Keywords:** Esophageal squamous cell carcinoma, tumor immune microenvironment, immune checkpoint inhibitors, immune predictive model, immunotherapy

## Abstract

Esophageal cancer is one of the most prevalent malignant tumors of the digestive tract, with esophageal squamous cell carcinoma (ESCC) as its main histological type. In recent years, immunotherapeutic regimens centered on immune checkpoint inhibitors (ICIs) have achieved remarkable efficacy in patients with advanced ESCC. However, due to the heterogeneity of the tumor immune microenvironment (TIME), the degree of clinical benefit varies among patients. This heterogeneity not only affects the biological behavior of tumors but also determines the efficacy and prognosis of immunotherapy. Therefore, exploring the interactions between tumor cells and diverse immunological components inside the TIME in ESCC, as well as how to use ICIs to reshape the TIME, may bring new therapeutic opportunities for ESCC patients. This review looks at the important aspects of TIME and immunological prediction models and explores potential therapeutic strategies targeting both, providing a theoretical basis for improving immunotherapy outcomes in ESCC.

## 1. Introduction

Surgery, radiation, chemotherapy, and targeted therapy are the standard treatments for ESCC.^1^ Survival prospects for individuals with advanced ESCC have improved dramatically as neoadjuvant immunotherapy—a combination of immunotherapy medicines, radiation, and chemotherapy—has become more widely used in treatment. However, only a tiny percentage of patients show a persistent clinical response to ICIs.^2^ As a result, immunotherapy research and the creation of tailored treatment options have become increasingly important. The TIME is a complex dynamic network that is directly linked to tumor start and progression. It includes not only tumor cells but also immune cell infiltration, immunosuppressive factor expression, and extracellular matrix remodeling. These characteristics all have an impact on how immunotherapy works.^3-5^ Additionally, aberrant expression of immunological checkpoints such as PD-1/PD-L1 and CTLA-4 is linked to tumor progression and a poor prognosis.^6^ As a result, learning more about TIME's dynamic alterations and discovering critical biomarkers for predicting ICI success will help researchers develop more effective immunotherapy regimens. This review highlights current studies on the compositional properties of the TIME in ESCC, as well as the creation of immunological prediction models.

## 2. The tumor immune microenvironment of esophageal squamous cell carcinoma

**Figure 1.**
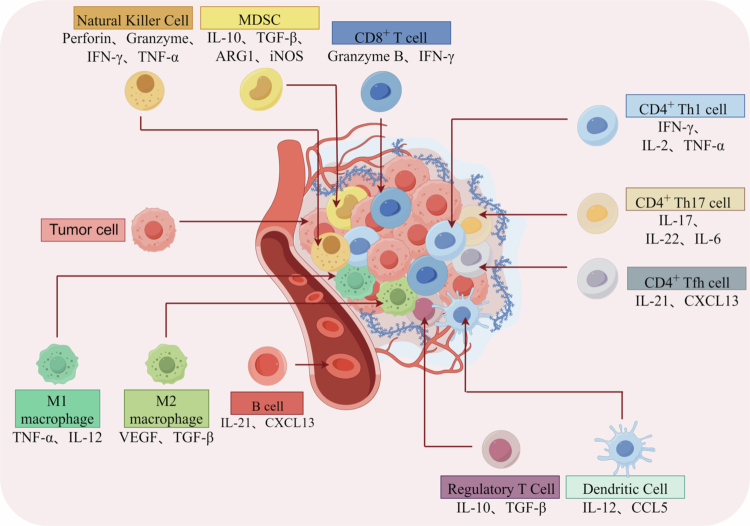
Classification of immune cells in the tumor immune microenvironment. Immune cells mainly include CD4^+^ T cells, CD8^+^ T cells, B cells, dendritic cells, natural killer cells, macrophages, regulatory T cells, and MDSCs. By secreting related cytokines, immune cells exert distinct protumor and antitumor effects in the tumor immune microenvironment.

The TIME in ESCC creates an immunosuppressive microenvironment with distinct inhibitory properties.^7^ As a core component, abnormalities in the composition, phenotype, and functional status of immune cells are key factors driving the formation of this immunosuppressive phenotype. In-depth exploration of the regulatory mechanisms of immune cells in ESCC is of great significance for understanding tumor growth, metastasis, and treatment response^8^ (Figure 1).

### 2.1. Tumor-infiltrating lymphocytes (TILs)

TILs are a prominent component of immune cells in the TIME, consisting of several unique cell types, such as cytotoxic T cells (CD8^+^ T cells), helper T cells (CD4^+^ T cells), B cells, dendritic cells (DCs), natural killer (NK) cells, and others. These cell types have distinct protumor and antitumor impacts in TIME, and their activation states and spatial distribution characteristics within tumors may differ as well.^9^

A growing body of research has demonstrated that high levels of TILs are related to a better prognosis in individuals with ESCC. CD8^+^ T cells serve as key effector cells in antitumor immunity, killing target cells directly by attaching to antigen-presenting molecules on tumor cell surfaces, specifically major histocompatibility complex class I (MHC-I) molecules. Li et al. discovered that the infiltration density of CD8^+^ T lymphocytes correlates with overall survival (OS) in patients with ESCC and that high levels of CD8^+^ T cells give a survival benefit to ESCC patients.^10^ However, this study quantified only the overall infiltration density of intratumoral CD8⁺ T cells without explicitly distinguishing whether these cells are localized in the stroma or intraepithelial region. If CD8⁺ T cells with high density in the stroma fail to infiltrate into the tumor parenchyma, they will have difficulty fully exerting their antitumor cytotoxic function. Therefore, the exclusion of CD8⁺ T cells from the tumor core region is one of the important mechanisms underlying immune escape in ESCC. Furthermore, during TIME, CD4^+^ T cells develop into numerous subtypes (including Th1, Th2, Th17, Tregs, and so on), which regulate the activity and function of other immune cells by secreting cytokines. Although immune cells are well known for their anticancer properties, Tregs—a specific type of helper T cell—typically perform immunosuppressive roles. They inhibit the proliferation and cytotoxic activity of CD8^+^ T cells, thereby promoting tumor immune escape, either by secreting cytokines such as IL-10 and TGF-β or through direct binding to effector T cells. Studies have demonstrated that Tregs in ESCC patients had increased immunosuppressive capabilities after radiation,^11^ and the presence of highly suppressive circulating Tregs may represent an immunosuppressive microenvironment (IME) induced by chemoradiotherapy. Therefore, therapies targeting Tregs or their immunosuppressive effector molecules (such as TGF-β and CTLA-4) may be key to reversing immune suppression.

In contrast, B cells in the TIME can impact malignancies by generating particular antibodies, presenting antigens, and secreting cytokines.^12^ According to research, the expression levels of B cells and B cell-related genes in TIME are highly correlated with treatment success in ESCC. Patients who received clinical benefit showed upregulation of genes related to B cells, B cell function, and plasma cells (all *p* < 0.05).^13^ In addition, DCs also play a vital role in the immune response. Within the TIME, they are capable of recognizing and presenting antigens to activate T cells. Consequently, DCs are considered a prospective target for activating the immune system. Nishimura et al. found that mature LAMP3^+^ DCs are related to more invading CD8^+^ T lymphocytes in ESCC.^14^ Notably, in other gastrointestinal tumors, LAMP3⁺ DCs can also promote immune suppression by recruiting Tregs or secreting immunosuppressive cytokines. Future studies should further investigate whether LAMP3⁺ DCs derived from ESCC possess similar dual functions. It remains controversial whether their correlation with CD8⁺ T cells truly facilitates antitumor immunity or is instead offset by concurrent immunosuppressive effects.

NK cells are innate immune cells found throughout the peripheral lymphoid organs and the circulatory system. They can identify and kill tumor cells by producing cytotoxic chemicals such as perforin and granzyme.^15^ NK cells secrete cytokines, including IFN-γ and TNF-α, which regulate immune responses and boost the actions of other immune cells.^16^ As early as 2000, scientists such as Imai hypothesized that populations with low NK cell activity are more prone to tumor formation, underlining the relevance of NK cells in antitumor immunity within the innate immune system.^17^ As our understanding of TIME grows, extensive scientific evidence suggests that NK cells may play a critical role in immune surveillance and antitumor responses in ESCC. Han et al. discovered that the percentage of CD16^+^ NK cells in ESCC tumor tissues was lower than that in normal esophageal mucosa. Combining radiation and chemotherapy with immune checkpoint blockade (ICB) treatment promoted CD16^+^ NK cells.^18^ Lim et al. indicated that in ESCC, NK cell infiltration within tumor tissues corresponds with a better prognosis. Expanded NK cells exhibit significant cytotoxic effects against ESCC cells expressing NKG2DL, indicating strong theoretical support for their clinical use in ESCC.^19^ Leveraging the unique innate immune regulatory capabilities of NK cells, CAR-NK cell therapy and new treatment techniques based on in vitro-grown and activated NK cells are now being investigated,^20^^,^^21^ NK cells are becoming a popular target for tumor treatment.

Overall, TILs play an important role in the occurrence and progression of ESCC. Different TILs serve unique activities in the TIME. A thorough examination of their kinds and functional activities provides critical insights for understanding tumor immunity and designing precision immunotherapy approaches.

### 2.2. Tumor-associated macrophages (TAMs)

TAMs are essential immune cells in the TIME. They are commonly seen in ESCC tumor tissues, and their quantity is strongly correlated with tumor invasiveness and prognosis. Under the influence of the microenvironment, macrophages can differentiate into two kinds based on their microenvironment: M1 and M2.^22^ Among these, the M1 phenotype has antitumor capabilities, secreting proinflammatory chemicals (such as TNF-α and IL-12), which decrease tumor growth by boosting immune responses and destroying tumor cells. In contrast, M2 macrophages promote tumor growth by secreting immunosuppressive substances, including IL-10 and TGF-β. This suppresses antitumor immune responses and increases tumor cell invasion. M2 macrophages can promote tumor growth and metastasis by secreting proangiogenic substances such as vascular endothelial growth factor (VEGF), which supplies nutrients and oxygen to malignancies. Furthermore, matrix metalloproteinases (MMPs) released by TAMs break down the extracellular matrix, allowing tumor cells to cross the stroma and enter the blood or lymphatic system, increasing metastasis. These actions make TAMs viable therapeutic targets, and TAM-targeted therapies may improve tumor therapy outcomes.^23^ In a recent study, Liu et al. developed a risk prediction model for ESCC based on prognostic genes. The study found a positive association between the risk score and the ratio of M1 to M2 macrophages. M2 macrophages showed a stronger correlation (*r *= 0.391, *p* < 0.001).^24^ Guo et al. also analyzed transcriptome data from esophageal dysplasia and ESCC tissues to look for changes in the TIME between the two states. The study found that ESCC had more M2 macrophages in TIME compared to precancerous esophageal dysplasia (*p* < 0.05), indicating that dynamic alterations in TIME may contribute to the development from esophageal dysplasia to ESCC.^25^ These findings indicate that the quantity and phenotypic characteristics of TAMs may serve as potential biomarkers for ESCC prognosis. Future research and therapy methods will focus on effectively targeting and manipulating TAMs to improve clinical outcomes and treatment efficacy in ESCC patients.

### 2.3. Myeloid-derived suppressor cells (MDSCs)

In ESCC, MDSCs act as key immunosuppressive cells and exert their immunosuppressive functions through multiple mechanisms, including inhibiting the proliferation and activation of T cells, stimulating tumor angiogenesis, and promoting the invasion and metastasis of tumor cells. MDSCs are typically classified into two subtypes based on their distinct immunophenotypes: granulocyte-like MDSCs (G-MDSCs) and monocyte-like MDSCs (M-MDSCs),^26^^,^^27^ respectively, suppressing T cell activity through the arginine metabolic pathway (ARG1 and iNOS synthesis) and by secreting immunosuppressive substances, including IL-10 and TGF-β.^28^ As reported by Karakasheva et al., CD38 plays a critical role in promoting the expansion of M-MDSCs and regulating the expression of iNOS. It facilitates the progression of ESCC by inhibiting T cell-mediated immune responses.^29^ Similarly, Wen et al. discovered that MDSCs that overexpress Lnc-17Rik increase ESCC tumor formation by improving their immunosuppressive activities.^30^ Although the foregoing research findings show that MDSCs' immunosuppressive activity is important in the formation and progression of ESCC, several limitations and controversies persist. For instance, analyzing MDSCs as a homogeneous population may mask the heterogeneity of the two distinct subsets in response to markers. Furthermore, phenotypic and functional discrepancies exist between in vitro-induced MDSCs and tumor-in situ-recruited MDSCs, potentially compromising the accuracy of evaluating MDSC immunosuppressive function. Future research should be further refined through validation in human samples, dynamic microenvironment analysis, and the development of targeted intervention strategies, thereby providing a new research technique for immunotherapy in ESCC patients.

## 3. Immunotherapy efficacy prediction model for esophageal squamous cell carcinoma

**Figure 2.**
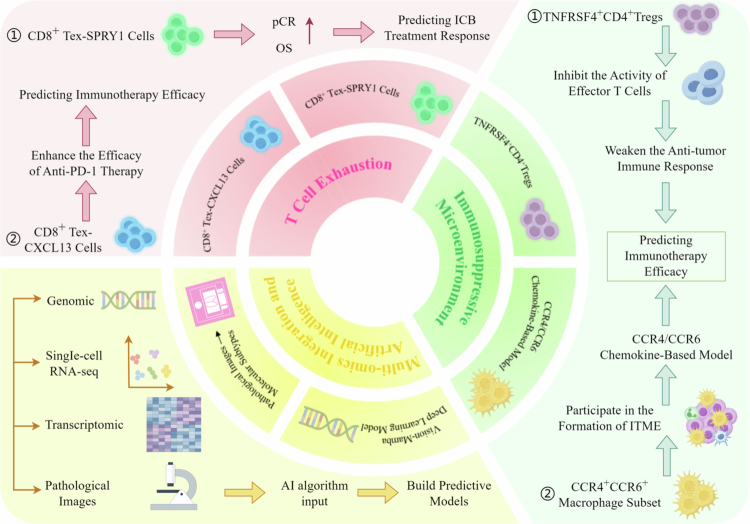
Prediction models for immunotherapy efficacy. Prediction models mainly include predictive model for T cell exhaustion state, predictive model for the immunosuppressive microenvironment, and prediction models based on multiomics integration and artificial intelligence. By integrating multiomics data, the accuracy of model prediction has improved, and a new theoretical basis has been provided for personalized treatment decisions.

Although neoadjuvant immunotherapy has shown great efficacy in ESCC, there is significant inter-patient variability in treatment response. As a result, researchers are currently focusing on developing more precise models for predicting treatment outcomes. Currently, prediction models based on single biomarkers such as PD-L1 expression levels, tumor mutational burden (TMB), and microsatellite instability (MSI) are of poor therapeutic usefulness. Next-generation integrated models are using multiomics data, such as genetic and transcriptomic markers, as well as immunological microenvironment factors, to improve prediction efficacy. This strategy not only improves model predictions but it also establishes a new theoretical foundation for personalized treatment decisions^31^ (Figure 2).

### 3.1. Predictive model for T cell exhaustion state

CD8^+^ T cells serve as central effector cells in antitumor immune responses, directly recognizing and eliminating tumor cells through differentiation into cytotoxic effector T cells. However, CD8^+^ T cells frequently reach a state of fatigue due to functional loss, reducing their antitumor potency.^32^ To address this issue, targeted blockade therapies against immune checkpoint molecules such as PD-1/PD-L1 can reverse T-cell exhaustion and restore their antitumor activity. Advances in single-cell sequencing technologies have revealed that dynamic changes in the phenotype and function of certain immune cell subsets within the TIME are important regulators of treatment response. Multiple studies have shown that the CD8^+^ T cell subset with a progenitor-like exhausted phenotype is closely associated with the response of ESCC patients to neoadjuvant immunotherapy. Liu et al. used single-cell RNA sequencing (scRNA-seq) to identify a population of exhausted CD8^+^ T cells (CD8^+^ Tex-SPRY1) highly expressing SPRY1 in ESCC tumor tissues. The pretreatment enrichment level of this subpopulation is significantly correlated with pathological complete response (pCR) and improved overall survival in patients. Furthermore, in an independent ICB treatment cohort, this subset was validated as a specific biomarker with predictive value superior to conventional PD-L1 expression levels.^33^ Although this biomarker shows promising research potential, predictive models constructed based on single-cell transcriptomic data carry a risk of overfitting due to the high-dimensional nature of the data. Therefore, further validation in larger-scale, multicenter, prospective cohorts is required to rule out biases caused by cohort specificity or spurious associations. Another study on neoadjuvant immunochemotherapy (nICT) focused on exhausted CD8^+^ T cells (CD8^+^ Tex-CXCL13) that expressed high levels of CXCL13. These cells were highly infiltrated in tumor tissues of treatment responders and linked positively with the quantity of tertiary lymphoid structures (TLS) after therapy. Simultaneously, CXCL13 also increases the efficacy of anti-PD-1 treatment by attracting B cells and CD8^+^ T cells.^34^ Notably, this study was based solely on a small discovery cohort and lacked support from an independent validation cohort or external public datasets. Furthermore, the validation of biomarker specificity and stability remains inadequate. Future research should expand the cohort, conduct multicenter validation, and combine functional experiments to further elucidate the specific mechanisms of action of CXCL13, thereby enhancing the model's clinical translational potential.

Considering the heterogeneity between cancerous and noncancerous cohorts in clinical studies regarding baseline characteristics and confounding factors, as well as the availability of relevant high-quality studies, these studies practically adopted cell subsets compared between the high-expression and low-expression groups within tumor tissue cohorts of ESCC patients. This design is essentially consistent with the control definition of the PECO framework. In summary, based on the studies of the above cell subsets, it is speculated that a predictive model centered on progenitor-like exhausted CD8^+^ T cells may provide multidimensional potential targets for immunotherapy in ESCC patients. However, its validity and applicability still need to be further verified and improved by studies with larger sample sizes and multicenter designs. Future research should further explore the functional regulatory mechanisms of these immune cell subsets, thereby providing new directions for the precision treatment of ESCC.

### 3.2. Predictive model for the immunosuppressive microenvironment

During the initiation and progression of tumors, the IME plays a pivotal role. By establishing intricate immune evasion networks, it helps tumor cells evade the surveillance and attack of the immune system, thereby promoting tumor growth, invasion, and metastasis. In recent years, researchers have focused on constructing prediction models for IME characteristics in order to obtain deeper insights into tumor immune suppression processes and attain precise medicine. These models aim to assess cancer patients' immunological status in advance, providing a scientific foundation for customized treatment.^35^ Several studies on ESCC have revealed key cellular subpopulations in the IME and their regulation mechanisms. Yang et al. discovered that in ESCC patients undergoing nICT, the tumor microenvironment of nonresponders showed marked immunosuppressive features: TNFRSF4^+^ CD4^+^ Tregs underwent clonal expansion and enhanced immunosuppressive function, directly suppressing effector T cell activity and weakening the antitumor immune response. Concurrently, LRRC15^+^ cancer-associated fibroblasts (CAFs), which are concentrated in nonresponders, attract TNFRSF4^+^ Tregs via the LGALS9-CD44 axis. This creates a locally immunosuppressive milieu, further facilitating immune escape.^36^ Another study found a CCR4^+^ CCR6^+^ macrophage subpopulation that was considerably enriched in untreated ESCC tissues but significantly reduced after neoadjuvant chemoradiotherapy (nCRT). This subpopulation contributes to IME development via intricate intercellular interactions, which influence the efficiency of anticancer immune responses. Based on their findings, the researchers created a predictive model focused on the CCR4/CCR6 chemokine system. This model includes seven important genes and effectively assesses the efficiency of nCRT in combination with ICB. It provides potential biomarkers and practical tools for assessing the immune status of ESCC patients and formulating personalized treatment strategies.^37^ Notably, the quantitative synthesis of these studies was based on only a small number of meta-analyzable studies, and the study populations were restricted to relevant cell subpopulations in ESCC. Therefore, the statistical stability of the relevant results and their generalizability across different tumor types still require further validation by additional studies.

Extensive preclinical evidence provides a robust theoretical foundation for immunotherapy based on IME prediction models. Currently, several clinical trials investigating chemo-radiotherapy combined with immunotherapy for ESCC are underway. However, several critical issues remain unresolved in individualized treatment strategies based on IME predictive models. These include the risk of overfitting due to relatively limited sample sizes, which compromises performance in real-world clinical applications; inconsistencies in multiomics data analysis that affect model reproducibility; and the combined predictive value of these models with traditional clinicopathological indicators (such as tumor stage and differentiation grade). Therefore, the optimal paradigm for IME-based personalized therapy requires further exploration in additional clinical trials.

### 3.3. Prediction models based on multiomics integration and artificial intelligence

The rapid development of high-throughput sequencing and mass spectrometry technologies has provided strong technical support for multiomics research, allowing for the systematic analysis of tumor molecular characteristics at multiple levels, including genomics, transcriptomics, proteomics, and metabolomics. Comprehensive analysis based on multiomics data not only enables deeper insights into the pathogenesis and progression of ESCC but also helps identify potential biomarkers and novel therapeutic targets. Concurrently, techniques such as machine learning and deep learning in the field of artificial intelligence (AI) show distinct advantages in mining complex biomedical large data because of their powerful data processing capabilities. These algorithms may extract key information from multiomics data to create precise predictive models, providing critical evidence for early detection, prognostic assessment, and individualized therapy of ESCC.^38^ In related research, Jiang et al. divided ESCC into four subgroups using genomic, transcriptomic, single-cell data, and pathological pictures. By applying deep learning models, they demonstrated the potential of pathological images to predict molecular characteristics, achieving automated mapping from pathological images to molecular subtypes. This work lays the groundwork for future AI-assisted diagnostic systems. This multiomics-integrated molecular typing technique more accurately reflects tumor heterogeneity, providing new insights for individualized therapeutic treatment.^39^ On the other hand, Zhang et al. created a deep learning model called Vision-Mamba. This model, which analyzes CT image features, can reliably predict the pCR status of ESCC patients following nICT, offering strong theoretical support for optimizing clinical treatment options.^40^

In conclusion, comprehensive analysis of ESCC molecular characteristics using multiomics technologies, combined with AI algorithms for efficient processing and analysis of complex multiomics data, has yielded promising applications in developing models for prognosis prediction, treatment response prediction, and early diagnosis prediction. However, this discipline continues to encounter various hurdles, including data quality management, algorithm optimization, insufficient model cohort sizes, and a lack of clinical validation. Future research should promote interdisciplinary collaboration, continuously refine technological approaches and research tactics, and promote the translation of relevant findings into clinical practice to deliver better therapeutic efficacy and survival outcomes for patients with ESCC (Table 1).

**Table 1.** Immunotherapy efficacy prediction model for ESCC.

Prediction model	Key biomarkers	Clinical applicability	Strengths	Limitations
T cell exhaustion state	CD8^+^ Tex-SPRY1 CD8^+^ Tex-CXCL13	Immunotherapeutic efficacy evaluation	Clear mechanisms and favorable clinical translation	Specificity and stability of biomarkers
Immunosuppressive microenvironment	TNFRSF4^+^ CD4^+^ Tregs	Tumor progression and prognosis	Evaluation of tumor malignancy and metastatic potential	Reproducibility and overfitting risk
Multiomics integration and artificial intelligence	Vision-Mamba	Comprehensive efficacy and resistance mechanisms	High precision and strong anti-interference capability	Data control and lack of clinical validation

## 4. Advances in immunotherapy for esophageal squamous cell carcinoma

**Figure 3.**
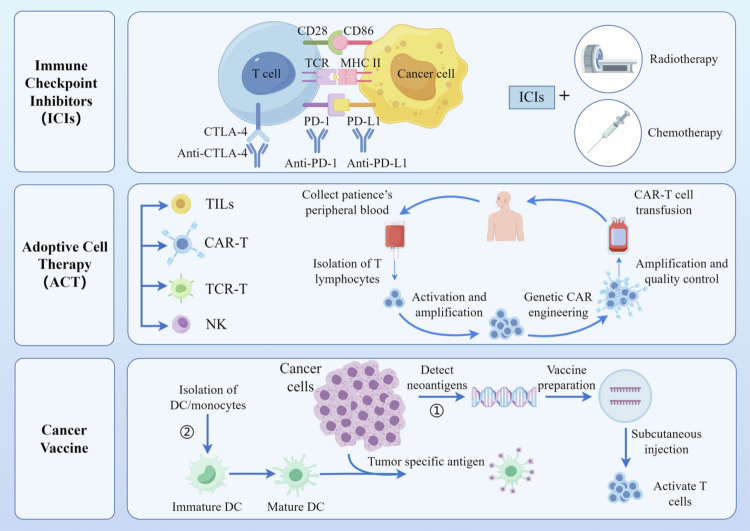
Advances in immunotherapy. The immunotherapy for esophageal squamous cell carcinoma mainly focuses on three aspects: immune checkpoint inhibitors, adoptive cell therapy, and cancer vaccines.

In recent years, research targeting tumor immune escape and immune tolerance mechanisms has advanced the development of neoadjuvant immunotherapy. Immune escape is a key hallmark of cancer, enabling tumor cells to resist immune cell attacks through multiple mechanisms, thereby promoting tumor growth and metastasis (Figure 3). This tumor–host immune relationship is termed the “3E model,” encompassing three stages: elimination, equilibrium, and escape.^41^ This model describes how the host immune system dynamically eliminates malignant cell precursors and controls micrometastases until tumor cells acquire genetic or epigenetic alterations, thereby achieving immune escape.^42^ The recent introduction of the “3C model,” concept has further enriched our understanding of key characteristics of cancer immune evasion. By describing the mechanisms through which tumor cells evade immune surveillance across three stages—camouflage, coercion, and cellular protection—it offers promising strategies for modern immunotherapy.^43^

### 4.1. Application of immune checkpoint inhibitors

ICIs have been the subject of several clinical trials to date, with the goal of lowering the tumor burden by strengthening patients' immune systems with ICI preoperative administration. This strategy aims to reduce the risk of postoperative recurrence and increase surgical resection rates.^44^^,^^45^ These investigations cover a range of ICIs, such as CTLA-4 inhibitors, TIM-3 inhibitors, LAG-3 inhibitors, and PD-1/PD-L1 inhibitors.^46^ The ESCC has focused heavily on PD-1/PD-L1 inhibitor research. These medicines keep T cells active and boost the immune system's ability to attack tumor cells by inhibiting inhibitory signals between tumor cells and T cells. They play an important role in overcoming tumor immune escape and tolerance. Cao et al. conducted a Phase III clinical trial (KEYNOTE-181) that found pembrolizumab to be a safe and effective second-line treatment for ESCC patients. The study found that PD-L1 CPS ≥1 was an excellent biomarker for predicting treatment success.^47^ Wang et al. further discovered that the coexpression of PD-L1/TIM3 and PD-L1/TIGIT is an independent prognostic factor for poor prognosis in patients with ESCC, which provides a theoretical basis for further exploring dual blockade therapy.^48^ Furthermore, CTLA-4 inhibitors improve immunotherapy efficiency by preventing CTLA-4 binding to its ligands CD80/CD86, which promotes T-cell activation and proliferation. Their application is also thought to play an important role in ESCC immunotherapy.^49^ Studies confirm that tremelimumab not only extends OS and progression-free survival (PFS) in ESCC patients, but it also improves therapeutic efficacy and reduces the incidence of adverse reactions when taken with PD-1 inhibitors.^50^ In conclusion, important findings from these varied tumor models jointly reveal ICIs' great therapeutic promise in ESCC treatment.

With ongoing developments in therapeutic methods, the combination of ICIs and chemoradiotherapy has shown excellent results in treating ESCC. Multiple studies indicate that chemoradiotherapy activates the immune system while reducing immunosuppressive cells within the TIME. ICIs boost T-cell antitumor efficacy by inhibiting immunological checkpoints such as PD-1/PD-L1 and CTLA-4. Combining these two modalities has a strong synergistic impact, increasing therapeutic efficacy.^51^ Clinical data reported by Xu et al. demonstrated that, when compared to standard chemotherapy regimens, the treatment group receiving camrelizumab combined with paclitaxel and cisplatin had significantly better OS and PFS. This regimen has the potential to establish a new standard for first-line treatment in patients with advanced or metastatic ESCC.^52^ Subsequent research by Luo et al. further validated the clinical value of this combination therapy, showing its potential to considerably improve treatment efficacy while keeping adverse effects within acceptable levels. These data significantly support its potential use in clinical practice.^53^

However, the application of ICIs, especially the implementation of combined immunotherapy strategies, has brought breakthrough progress in ESCC treatment while also being accompanied by a series of unique immune-related adverse events (irAEs). The most common side effects include skin toxicity, gastrointestinal reactions, endocrine disorders, and hepatic toxicity.^54^ Notably, while combined immunotherapy improves patients' survival benefits, it not only increases the overall incidence of irAEs but also elevates the risk of severe irAEs such as pneumonia, nephritis, and myocarditis.^55^ Therefore, in the clinical application of ICI-based combined immunotherapy regimens, it is necessary to strengthen the whole-course monitoring of patients, promptly identify and intervene in relevant side effects, so as to balance treatment efficacy and safety.

### 4.2. Adoptive cell therapy (ACT)

ACT, as an emerging immunotherapeutic approach, focuses on the in vitro expansion and genetic modification of a patient's own immune cells to enhance their antitumor activity. These modified cells are then reinfused back into the patient, boosting the body's immunological response to malignancies. Compared to conventional therapies, ACT offers distinct advantages, such as high targeting specificity and durable immune memory, providing a novel treatment option for patients with clinically refractory ESCC.^56^ Several treatment techniques are currently being tested in clinical trials, including tumor-infiltrating lymphocyte (TIL) therapy, chimeric antigen receptor T-cell (CAR-T) therapy, T-cell receptor-engineered T-cell (TCR-T) therapy, and NK cell therapy.^57^ Among these techniques, CAR-T therapy has emerged as a recent research focus. It uses genetic engineering to modify T lymphocytes to express chimeric antigen receptors (CARs), which greatly improves their ability to recognize and kill tumor cells. CLDN18.2-specific CAR-T cells (CT041) have demonstrated significant efficacy in gastrointestinal cancers, achieving an ORR of 48.6% in advanced ESCC patients.^58^ Furthermore, studies show that CAR-T cells targeting MUC1 successfully kill ESCC cells in vitro and have increased proliferative potential and long-lasting anticancer effects in mice models, notably subcutaneous xenograft tumors and humanized PDX models.^59^ In contrast, research findings on TILs and TCR-T treatment are rare, with clinical trials remaining in the exploratory stage. Although ACT has potential in ESCC treatment, tumor heterogeneity and the immunosuppressive microenvironment remain important factors influencing efficacy. Future studies will concentrate on optimizing cell preparations, improving tumor targeting, and combating immune escape mechanisms.

### 4.3. Cancer vaccine

With ongoing research, immunotherapy regimens for ESCC based on tumor vaccines have made substantial progress. Several vaccine forms, including peptide vaccines, DC vaccines, and neoantigen vaccines, have shown efficacy in clinical studies. In recent years, researchers have discovered multiple immunogenic cancer antigens (ICAs) that are overexpressed in ESCC cells. These ICAs also play critical roles in tumor cell survival and proliferation, making them ideal targets for ESCC tumor vaccines.^60^^,^^61^ In preliminary research, Kosaku et al. found three HLA-A24-restricted immunodominant peptides (derived from TTK, LY6K, and IMP3, respectively) and conducted Phase I/II clinical trials of combination vaccination containing these three peptide groups. The data indicated that patients who developed an immune response to the peptide vaccine demonstrated a better prognosis compared to those without an immune response.^62^ These findings prompted further research. Currently, two clinical trials using LY6K, CDCA1, and KOC1 peptide vaccines are underway to evaluate their clinical value as postoperative adjuvant therapies.^63^ Given that DCs serve as the primary antigen-presenting cells and activators of T cells, several research have focused on the application of peptide-pulsed DCs as cellular vaccines. A clinical experiment conducted by Narita et al. found that while SART1 peptide-pulsed DC vaccination did not significantly improve patient clinical outcomes, it had a good tolerability and safety profile.^64^ Additionally, DC vaccines pulsed with WT1 peptides have been shown to boost WT1-specific immune responses.^65^^,^^66^ Neoantigen vaccines are currently the most extensively studied types of tumor vaccines. Tumor-specific antigens (TSAs), also known as neoantigens, are formed as a result of the genetic instability of tumor cells, which contains multiple somatic mutations. Neoantigen-based mRNA vaccines stimulate the immune system by targeting TSAs, resulting in strong tumor-specific T-cell responses. Wang et al. recently published a case study of an ESCC patient who was treated with a tailored mRNA vaccination paired with a PD-1 inhibitor, resulting in partial remission and allowing the patient to live for 457 d with OS and PFS.^67^ This achievement not only confirms the feasibility of mRNA vaccines in ESCC for the first time but also provides the groundwork for optimizing future combination therapy techniques. In summary, research into tumor vaccine immunotherapy for ESCC is progressing, with numerous vaccine forms and combination treatment regimens being investigated. In the future, it is necessary to overcome limitations such as difficulties in vaccine design and the IME, providing new therapeutic hope for ESCC patients (Table 2).

**Table 2.** Efficacy outcomes and common side effects of immunotherapy in ESCC.

Immunotherapy for ESCC	Study	Patient population	Treatment outcomes	Complications
ICIs	Cao et al. [47]	401	ORR: experimental group (21.5%) Control group (6.1%)	Neutropenia hypothyroidism
	Xu et al. [52]	596	3-y OS rate: experimental group (25.6%) Control group (12.8%)	Anemia gastrointestinal reactions irAEs
ACT	Qi et al. [58]	37	Overall ORR: 48.6%	CRS Anemia
Cancer vaccine	Mimura K et al. [62]	60	Responders showed longer PFS and OS	Mild systemic inflammatory response
	Wang et al.[67]	1	The patient to live for 457 d with OS and PFS	Thrombocytopenia vaccine-related adverse events

## 5. Conclusion

The dynamic changes of immune cells in the TIME are a key factor regulating the efficacy of immunotherapy for ESCC. Immunotherapeutic regimens based on TIME have significantly improved patient survival outcomes and provided a new direction for personalized treatment. Furthermore, efficacy prediction models are evolving from single biomarkers toward multiomics integration and artificial intelligence, aiming to enhance predictive accuracy and maximize therapeutic outcomes.

Currently, the understanding of the molecular mechanisms by which TIME influences immunotherapy in ESCC remains limited. Prediction models also face limitations such as insufficient validation in different patient cohorts and the risk of overfitting in small datasets. Future studies on TIME could further explore additional biomarkers, regulatory factors, and signaling pathways while evaluating the specificity and variability of these molecules among different ESCC patients. It is crucial to deeply clarify the regulatory mechanisms underlying TIME heterogeneity in ESCC, screen robust and reproducible biomarkers that reflect immune function rather than merely cell quantity, and validate precise prediction models through multicenter, large-sample studies. Meanwhile, the integration of multiomics data, artificial intelligence, and longitudinal clinical data is essential for revealing environment-dependent immune regulatory networks and optimizing patient stratification strategies. By positioning TIME as a central entry point, efforts should be made to promote the translation of theoretical research on ESCC immunotherapy into clinical practice, optimize combined immunotherapy strategies, and ultimately achieve personalized precision treatment for ESCC to improve patient outcomes.

## Supplementary Material

Literature retrieval.docx Literature retrieval.docx

## Data Availability

Data sharing is not applicable to this article as no data were created or analyzed in this study.
