# Supplementary material for: Research progress on the compositional characteristics of the tumor immune microenvironment and immunopredictive models in esophageal squamous cell carcinoma
Source: Cancer Biol Ther. 2026 Mar 11;27(1):2641263. doi: 10.1080/15384047.2026.2641263 (PMC12987515; doi:10.1080/15384047.2026.2641263)
Supplement: Literature retrieval.docx [file KCBT_A_2641263_SM3390.docx]

The literature search databases for this review include PubMed, Embase, Cochrane Library and Web of Science, with the search time frame from the establishment of each database to December 31, 2025.

Search terms were designed around three core dimensions: esophageal squamous cell carcinoma, tumor immune microenvironment, and immune predictive model, encompassing both MeSH Terms and free-text terms (Title/Abstract). During the search process, Boolean operators (AND/OR/NOT) were used to combine search terms. Additionally, the reference lists of included studies were manually searched to supplement potentially missed literature.

Inclusion Criteria:

1. Study types: clinical studies, basic experimental studies, or meta-analyses.
2. Study content involving: esophageal squamous cell carcinoma; tumor immune microenvironment; immune checkpoint inhibitors; immune predictive models; immunotherapy.
3. Provision of complete raw data or extractable key results.
4. Language restriction: English.

Exclusion Criteria:

1. Case reports, conference abstracts, and literature without complete data.
2. Duplicate publications or studies of low quality.

Full electronic search strings for each database are provided as follows (example):

(("esophageal squamous cell carcinoma"[MeSH Terms]) AND ("tumor immune microenvironment"[MeSH Terms] OR "tumor microenvironment"[Title/Abstract] OR "immune microenvironment"[Title/Abstract]) AND ("immune predictive model"[Title/Abstract] OR "predictive biomarker"[Title/Abstract] OR "prognostic model"[Title/Abstract]) AND ("clinical study"[MeSH Terms] OR "basic experimental study"[Title/Abstract] OR "meta-analyses"[Publication Type])) NOT ("case report"[Publication Type] OR "conference abstract"[Publication Type]).
